# SOD1:c.118G>A mutation in the Cimarrón Uruguayo dog: prevalence, drift dynamics, and structural effects

**DOI:** 10.29374/2527-2179.bjvm005526

**Published:** 2026-09-11

**Authors:** Rody Artigas, Carolina Menchaca, Eugenio Jara, Alejandra Mondino, Noelia Vázquez, Andrea Branda Sica, Silvia Llambí

**Affiliations:** 1 Animal Breeding Academic Unit, Department of Animal Production and Health of Production Systems, Faculty of Veterinary Medicine, University of the Republic (Udelar), Montevideo, Uruguay.; 2 Center for Encephalopathies and Emerging Transmissible Diseases, University of Zaragoza (UNIZAR), Zaragoza, Spain.; 3 Department of Clinical Sciences, North Carolina State University, Raleigh, North Carolina, United States.; 4 Anatomy Academic Unit, Department of Biosciences, Faculty of Veterinary Medicine, University of the Republic (Udelar), Montevideo, Uruguay.; 5 National Institute of Agricultural Research (INIA), Las Brujas, Canelones, Uruguay.

**Keywords:** degenerative myelopathy, SOD1 mutation, genetic drift, electrostatic potential, mielopatia degenerativa, mutação SOD1, deriva genética, potencial eletrostático

## Abstract

Canine degenerative myelopathy (DM) is associated with a missense mutation in the *SOD1* gene (c.118G>A), which results in the E40K amino acid substitution and promotes protein aggregation mechanisms similar to those described in human amyotrophic lateral sclerosis. The present study estimated the frequency of the SOD1:c.118G>A mutation in the Cimarrón Uruguayo dog and evaluated its structural and population implications. A total of 82 dogs were genotyped. The mutant allele was detected at a frequency within the range of common variants (q = 0.177), and genotype distribution did not deviate from Hardy–Weinberg equilibrium. Wright–Fisher simulations showed that stochastic processes alone may substantially influence allele-frequency trajectories over time. Additionally, a high-resolution melting (HRM) assay was optimized and enabled clear discrimination of the three genotypes, supporting its use as a rapid genotyping approach. Sanger sequencing of SOD1 exons 2–5 was performed in a subset of individuals to identify additional coding variants. Sequencing revealed no additional non-synonymous variants, except for a synonymous substitution in exon 4. Structural inspection indicated that the E40K substitution does not alter the local hydrogen-bonding pattern but produces a charge inversion that modifies the electrostatic environment surrounding residues 40 and 91. These findings support the hypothesis that electrostatic alterations in this region may contribute to an increased aggregation propensity of mutant SOD1 and provide new insights into the molecular and population context of this mutation in the Cimarrón Uruguayo breed.

## Resumo

A mielopatia degenerativa canina (DM) está associada a uma mutação missense no gene SOD1 (c.118G>A), que resulta na substituição do aminoácido E40K e promove mecanismos de agregação proteica semelhantes aos descritos na esclerose lateral amiotrófica humana. O presente estudo estimou a frequência da mutação SOD1:c.118G>A no cão Cimarrón Uruguayo e avaliou suas implicações estruturais e populacionais. Um total de 82 cães foi genotipado. O alelo mutante foi detectado em uma frequência dentro da faixa de variantes comuns (q = 0,177), e a distribuição genotípica não diferiu do esperado sob equilíbrio de Hardy–Weinberg. Simulações de Wright–Fisher mostraram que processos estocásticos podem influenciar substancialmente as trajetórias de frequência alélica ao longo do tempo. Adicionalmente, um ensaio de high-resolution melting (HRM) foi otimizado e permitiu clara discriminação dos três genótipos, apoiando seu uso como uma abordagem rápida de genotipagem. O sequenciamento por Sanger dos éxons 2–5 do gene SOD1 foi realizado em um subconjunto de indivíduos para identificar variantes adicionais na região codificante. O sequenciamento não revelou variantes não sinônimas adicionais, exceto por uma substituição sinônima no éxon 4. A inspeção estrutural indicou que a substituição E40K não altera o padrão local de ligações de hidrogênio, mas produz uma inversão de carga que modifica o ambiente eletrostático ao redor dos resíduos 40 e 91. Esses achados sustentam a hipótese de que alterações eletrostáticas nessa região podem contribuir para um aumento da propensão à agregação da SOD1 mutante e fornecem novos insights sobre o contexto molecular e populacional dessa mutação na raça Cimarrón Uruguayo.

## Introduction

Canine degenerative myelopathy (DM) is a progressive, late-onset neurodegenerative disease that affects the spinal cord of adult dogs and causes gradual loss of voluntary motor function, and eventually paralysis and death. Since its initial description in German Shepherd dogs (Averill Junior, 1973), DM has gained relevance both for its clinical impact in veterinary medicine and for its value as a translational model for studying amyotrophic lateral sclerosis (ALS) in humans (Qi et al., 2019; Shino et al., 2019; Zhu et al., 2023). In both cases, the intracellular accumulation of superoxide dismutase 1 (SOD1) enzyme aggregates is a shared histopathological feature, implying common pathogenic mechanisms such as endoplasmic reticulum stress, mitochondrial dysfunction, and the misfolded protein response (Yokota et al., 2021; Wakayama et al., 2022).

The discovery of a point mutation in the *SOD1* gene: c.118G>A, responsible for replacing the glutamate residue with lysine at position 40 (E40K) of the protein, allowed a strong genetic correlation with DM to be established in multiple breeds (Awano et al., 2009). This mutation alters the structural stability of the protein, promoting the formation of toxic aggregates through mechanisms such as disruption of salt bridges (Kimura et al., 2019), aberrant exposure of cysteine residues (Shino et al., 2019), and the formation of disulfide-bonded oligomers (Wakayama et al., 2022). Although the classical model assumes autosomal recessive inheritance with incomplete penetrance, several studies have detected clinical manifestations in heterozygous individuals, suggesting greater complexity in phenotypic expression (Qi et al., 2019; Wininger et al., 2011). While the SOD1:c.118G>A mutation is strongly associated with canine degenerative myelopathy, its structural effects on the canine SOD1 protein remain incompletely understood.

At the epidemiological level, variable frequencies of the mutant allele have been reported in different breeds and geographical regions. However, there is a notable absence of studies focusing on native Latin American dog breeds. The Cimarrón Uruguayo, the only dog breed native to Uruguay officially recognized in 2017 by the Fédération Cynologique Internationale (FCI, https://www.fci.be/es/), represents a genetic reservoir of particular interest due to its small population base and potential vulnerability to hereditary diseases. In this context, it is important to characterize the presence and distribution of the SOD1:c.118G>A mutation in this breed, both to establish a baseline assessment and to help prevent its spread through genetic conservation strategies.

The objectives of this study are to estimate the allele and genotype frequencies of the SOD1:c.118G>A mutation in Uruguayan Cimarrón dogs, as well as to explore the variability of the *SOD1* gene in the evaluated individuals and the possible impact of the detected variants on the structural stability of the SOD1 protein.

## Materials and methods

### Study period and location

The study was conducted from September 2023 to September 2025 at the Facultad de Veterinaria, Universidad de la República (Udelar), Montevideo, Uruguay.

### Animals

Genomic DNA samples from 82 Cimarrón Uruguayo dogs originating from different regions of Uruguay were obtained from the Small Animal DNA Bank of the Unidad Académica de Mejora Animal, Facultad de Veterinaria.. The samples were collected within the five years preceding this study. The cohort included both males and females of different ages (range: 1–18 years; mean: 8.9 ± 3.9 years). Relatedness among sampled dogs was assessed primarily using available pedigree records. In addition, animals were selected from geographically distant regions of Uruguay to minimize the inclusion of closely related individuals. None of the dogs showed clinical signs compatible with degenerative myelopathy (DM) according to the phenotypic records available at the time of sample collection. The present study was performed exclusively using archived DNA samples; no live animals were handled, and no additional biological samples were collected for this research.

### Genotyping of the c.118G>A mutation

Genotyping of the c.118G>A mutation was carried out by PCR–RFLP. A fragment of the *SOD1* gene encompassing this mutation was amplified using primers previously described by Holder et al. (2014). PCR reactions were performed in a Multigene II thermal cycler (Labnet Inc., USA) in a final volume of 25 μL containing 50 ng of genomic DNA, 1 μL of each primer (10 pmol/μL), 12.5 μL of ImmoMix (Bioline, Australia), and nuclease-free water to complete the final volume. Cycling conditions consisted of an initial denaturation step at 95°C for 10 min, followed by 35 cycles of denaturation at 94°C for 40 s, annealing at 55°C for 30 s, and extension at 72°C for 1 min, and a final extension at 72°C for 10 min. PCR products were checked by electrophoresis on 1.5% agarose gels stained with GoodView™ (SBS Genetech Co., Ltd., China).

Restriction digestion of the PCR products was performed using the *AcuI* restriction enzyme. Digestion reactions were prepared in a total volume of 25 μL containing 10 μL of PCR product, 2.5 μL of the corresponding buffer (New England Biolabs, USA), 1 μL of *AcuI* enzyme (New England Biolabs), and ultrapure water. Samples were incubated overnight at 37°C, followed by enzyme inactivation at 65°C for 20 min. Genotypes were determined based on the electrophoretic patterns of the restriction fragments resolved on 1.5% agarose gels stained with GoodView™. The PCR–RFLP assay was previously validated in our laboratory (Artigas et al., 2024).

### Population analysis

Allelic and genotypic frequencies were estimated by direct counting. Deviation from Hardy–Weinberg equilibrium was assessed using Fisher's exact test implemented in the SHEsis software (http://shesisplus.bio-x.cn/SHEsis.html. Ninety-five percent confidence intervals (95% CIs) for allelic and genotypic frequencies were calculated using the Wilson score method (Wilson, 1927).

### Analysis of allele frequencies under genetic drift

Allele-frequency dynamics were simulated under a neutral Wright–Fisher model of genetic drift (Fisher, 1930; Wright, 1931) assuming no selection, mutation, or migration, using R version 4.5.2 (R Foundation for Statistical Computing, Vienna, Austria).

The initial allele frequency (q_0_) was set according to the allelic frequency of the SOD1:c.118G>A mutation estimated in the present study in the Cimarrón Uruguayo population. The effective population size was fixed at Ne= 56.9, as previously reported for the breed in Uruguay (Martínez et al., 2011). The number of gene copies per generation was defined as 2Ne. Allele-frequency changes across generations were modeled as binomial sampling processes according to:


$$q_{t+1}=\frac{X_{t}}{n}$$ (1)

where ***q_t_*** represents the allele frequency at generation ***t***, ***X_t_*** ∼ Binomial (n,qt​) represents the number of mutant allele copies sampled in generation ***t+1*** from ***n = 2Ne*** gene copies per generation. A total of 5000 independent replicate populations were simulated over 100 discrete generations to obtain stable estimates of allele-frequency dynamics while evaluating long-term genetic drift. Selection, mutation, and migration were not included in the simulations; therefore, the model assumes neutrality and random mating.

#### Estimation of percentile trajectories

To characterize the dispersion of allele-frequency trajectories over time, percentile bands were constructed from the replicate simulations. For each generation, the distribution of allele frequencies across populations was summarized by calculating the 5th, 50th (median), and 95th percentiles using the base R function *quantile()*.

#### Expected disease prevalence

Assuming Hardy–Weinberg equilibrium within each population, the expected proportion of affected individuals at generation *t* was calculated as:

q^2^_t_ (2)

Percentile trajectories for expected prevalence were obtained from the simulated distributions of q^2^_t_ across replicates.

#### Threshold exceedance probabilities

The probability that the mutant allele frequency exceeded predefined thresholds selected as progressively higher values (q ≥ 0.20, q ≥ 0.25, and q ≥ 0.30) was calculated at each generation as:

P (q_t_ ≥ c) (3)

where *c* denotes the threshold value.

#### Software and packages

All simulations were performed using pseudo-random number generation with a fixed seed *(set.seed(123))* to ensure reproducibility. Data processing and visualization were performed using base R, dplyr (v1.2.0), tidyr (v1.3.2), and ggplot2 (v4.0.2) (Wickham, 2026a; Wickham, 2026b).

### Primer design for HRM analysis

Primers for HRM analysis were designed using Primer-BLAST (NCBI) to amplify a 79 bp fragment encompassing the SOD1:c.118G>A mutation (Supplementary Material Table 1). The design was based on the reference sequence of the canine SOD1 gene retrieved from Ensembl (Canis lupus familiaris, assembly ROS_Cfam_1.0). Primer specificity was assessed in silico using BLASTn against the canine genome.

**Table 1:** Genotypic and allelic frequencies for the SOD1:c.118G>A mutation in Cimarrón Uruguayo dogs.

**Genotype**	**N**	**Frequency**	**95% CI**
GG	56	0.683	0.576-0.774
GA	23	0.280	0.195-0.386
AA	3	0.037	0.013-0.102
	**Allele**		**95% CI**
G	0.823		0.758-0.874
A	0.177		0.126-0.242

### Optimization of genotyping by real-time PCR-HRM and data analysis

A real-time PCR assay followed by high-resolution melting (HRM) analysis was optimized for the detection of the SOD1:c.118G>A mutation. Amplifications were carried out using genomic DNA samples in a Rotor-Gene™ real-time PCR system (Corbet Life Science, Australia). PCR reactions were performed in a final volume of 25 μL containing 50 ng of genomic DNA, 12.5 μL of 1× HRM PCR Master Mix (Type-it® HRM PCR Kit, Qiagen), forward and reverse primers at a final concentration of 0.5 μM each, and nuclease-free water to reach the final volume. No template controls (NTCs) were included in each PCR reaction.

Assay optimization was performed using seven Cimarrón Uruguayo dogs previously genotyped by PCR-RFLP, representing the three possible genotypes (two AA, two GA, and three GG). Each sample was amplified in three independent PCR reactions. The amplification program consisted of an initial denaturation step at 95 °C for 10 min, followed by 40 cycles of denaturation at 95 °C for 5 s, annealing at 60 °C for 25 s, and extension at 72 °C for 20 s. After amplification, samples were subjected to two holds at 95 °C for 10 s and 45 °C for 1 min. Subsequently, high-resolution melting analysis was performed from 79 to 82 °C, with temperature increments of 0.1 °C and a hold of 2 s at each step. Fluorescence acquisition was performed in the green detection channel of the instrument (excitation 470; detection 510 nm). HRM curve analysis was conducted using Rotor-Gene Q software (version 2.3.1), allowing the discrimination of the three possible genotypes (GG, GA, and AA) based on normalized melting curves and difference plots.

### Amplification and sequencing of SOD1 gene exons

Ten randomly selected dogs were used for sequencing exons 2 to 5 of the *SOD1* gene. Target regions were amplified by PCR using primers previously described by Awano et al. (2009). PCR reactions were carried out in a Multigene II thermal cycler (Labnet Inc., USA) in a final volume of 25 μL containing 50 ng of genomic DNA, 1 μL of each primer (10 pmol/μL), 12.5 μL of ImmoMix (Bioline, Australia), and nuclease-free water to reach the final volume. Thermal cycling conditions consisted of an initial denaturation at 95°C for 10 min, followed by 35 cycles of denaturation at 94°C for 40 s, annealing at the primer-specific temperature (Supplementary Material Table 1) for 30 s, and extension 72°C for 1 min, followed by a final extension at 72°C for 10 min. PCR products were visualized by electrophoresis on 1.5% agarose gels stained with GoodView™ (SBS Genetech Co., Ltd., China). Amplicons were purified and sequenced in both directions by Macrogen Inc. (Seoul, South Korea).

### Variant analysis of the SOD1 gene

For each animal and exon, forward and reverse-complement reads were aligned using the ClustalW algorithm implemented in BioEdit v7.2.5 (Hall, 1999). Chromatograms were manually inspected to resolve base-calling discrepancies and to confirm heterozygous positions. Based on strand concordance, a bidirectional consensus sequence was generated for each individual. These consensus sequences were subsequently aligned against the reference *SOD1* coding sequence (NM_001003035.1) and the Ensembl *SOD1* sequence from the Canis lupus familiaris assembly ROS_Cfam_1.0 to identify variants within the coding region of the gene.

### In silico structural impact of SOD1 polymorphisms and amyloidogenic propensity

The structural impact of the non-synonymous SNPs on the protein was evaluated using Swiss-PdbViewer v4.1 (https://spdbv.vital-it.ch/), based on the canine SOD1 X-ray diffraction structure (PDB ID: 7WX0; 1.40 Å resolution) corresponding to the UniProtKB entry Q8WNN6. Hydrogen bond identification was performed according to the distance criteria described by Kim et al. (2020), considering ranges of 1.2–2.76 Å (with hydrogens present) and 2.195–3.3 Å (without hydrogens). Surface electrostatic potential was calculated using the Poisson–Boltzmann equation, as described by Jo et al. (2022), in order to assess possible alterations in charge distribution and their potential effects on protein structural stability and intermolecular and intramolecular interactions (Tomasi, 1981).

## Results

### Detection of the SOD1:c.118G>A mutation and population genetic analysis

**Figure 1:**
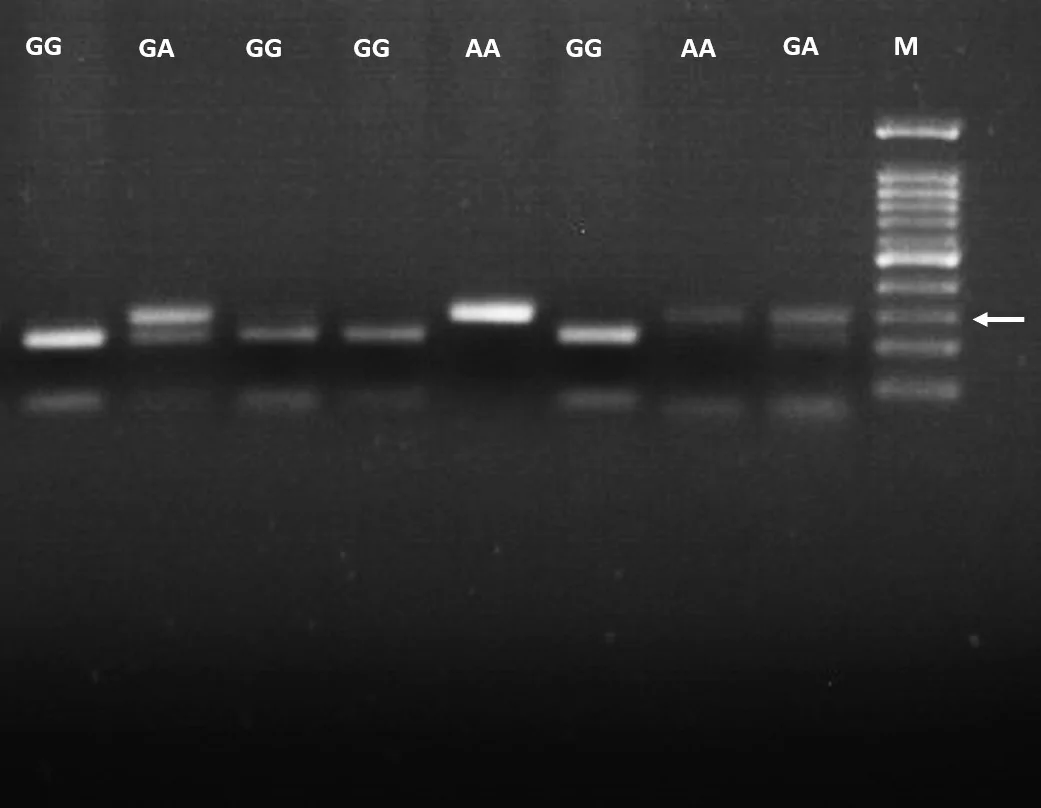
Restriction fragment patterns obtained after AcuI digestion of the SOD1:c.118G>A locus visualized on a 1.5% agarose gel. GG: homozygous wild-type genotype; GA: heterozygous genotype; AA: homozygous mutant genotype associated with increased susceptibility to degenerative myelopathy. M: 100 bp DNA ladder. The white arrow marks the 300 bp band of the marker.

PCR amplification generated a 292 bp fragment which, after digestion with the restriction enzyme *AcuI*, produced three distinct electrophoretic patterns. Homozygous mutant individuals (AA) displayed an undigested 292 bp fragment, heterozygous dogs (GA) showed three fragments (292 bp, 230 bp, and 62 bp), whereas homozygous wild-type animals (GG) exhibited two fragments of 230 bp and 62 bp (figure 1).

Genotypic and allelic frequencies of the SOD1:c.118G>A mutation in Cimarrón Uruguayo dogs are presented in Table 1. The wild-type GG genotype was the most frequent in the analyzed population, and the genotype distribution did not deviate from Hardy–Weinberg equilibrium (p=0.74). The mutant A allele was detected at a frequency of 0.177 in the studied population.

### Analysis of allele frequencies under genetic drift

**Figure 2:**
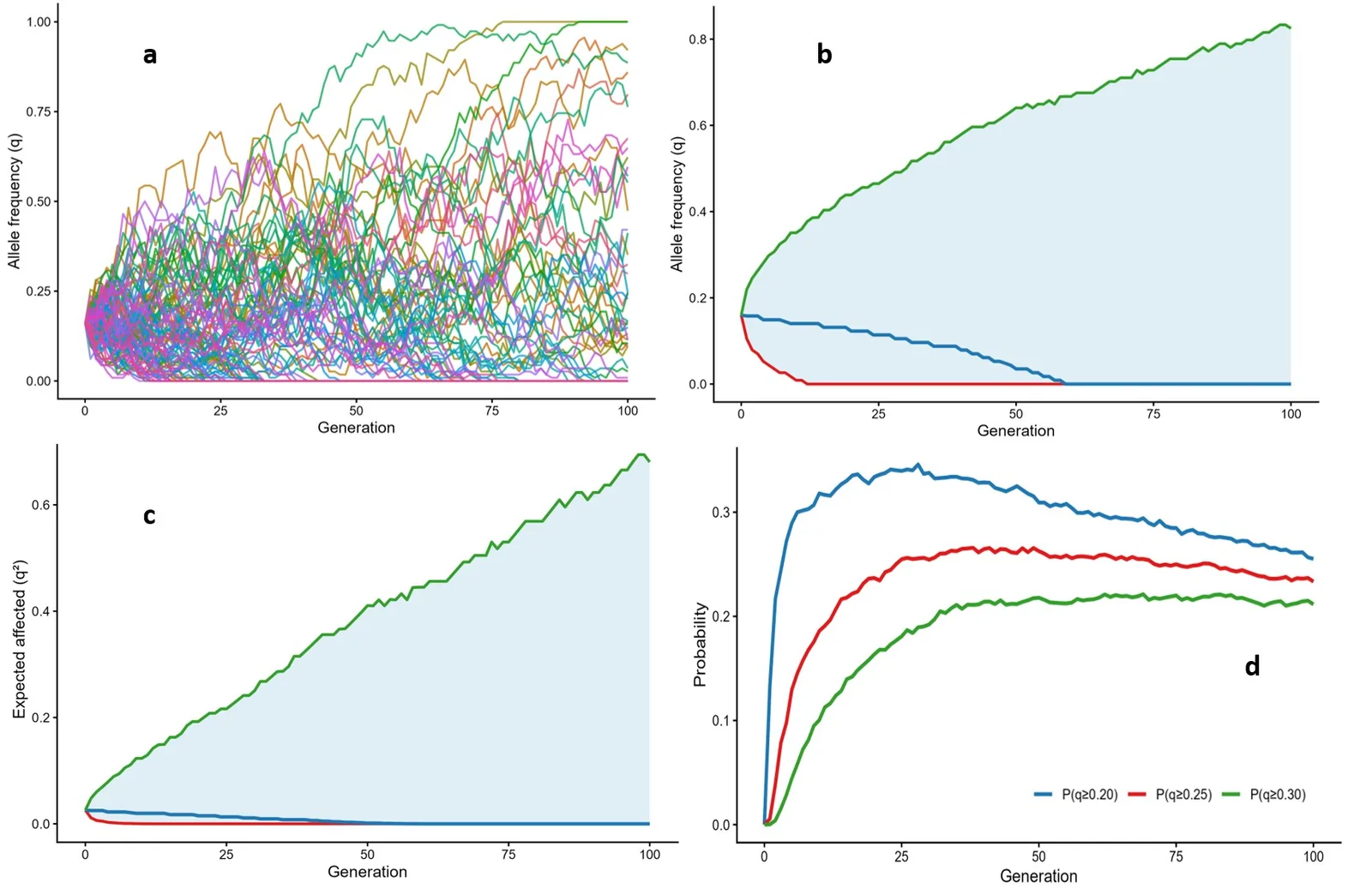
Wright–Fisher genetic drift simulations for the SOD1:c.118G>A allele in the Cimarrón Uruguayo population. (a) Individual allele-frequency trajectories (q) across 100 generations obtained from 100 replicate simulated populations. (b) Dispersion of allele-frequency trajectories summarized by percentile bands across simulations, showing the 5th (P5), 50th (P50), and 95th (P95) percentiles of q over time. (c) Percentile bands for the expected proportion of affected individuals (q^2^) derived from simulated allele frequencies, showing P5, P50, and P95 across generations. (d) Probability over time that allele frequency exceeds selected thresholds (q ≥ 0.20, q ≥ 0.25, q ≥ 0.30) estimated across replicate simulations.

Allele-frequency trajectories simulated under pure genetic drift are shown in figure 2**.** Individual replicate populations (figure 2a) exhibited marked stochastic divergence despite identical initial conditions (q_0_=0.177; Ne = 56.9), with outcomes ranging from allele loss to near fixation within 100 generations.

Percentile dynamics **(**figure 2b**)** revealed a rapid dispersion among replicate populations: the 5th percentile reached allele loss within approximately 15 generations, whereas the median trajectory declined to zero around generation 60. In contrast, the upper percentile increased steadily, exceeding q = 0.8 by generation 100.

When expressed as expected disease prevalence (q^2^; figure 2c), trajectories became strongly asymmetric. Although the median prevalence approached zero, the upper percentile increased markedly over time.

The probability of exceeding predefined allele-frequency thresholds (figure 2d) peaked between generations 20 and 40, after which it progressively declined as an increasing number of populations reached allele loss.

### HRM-based discrimination of SOD1:c.118G>A genotypes

**Figure 3:**
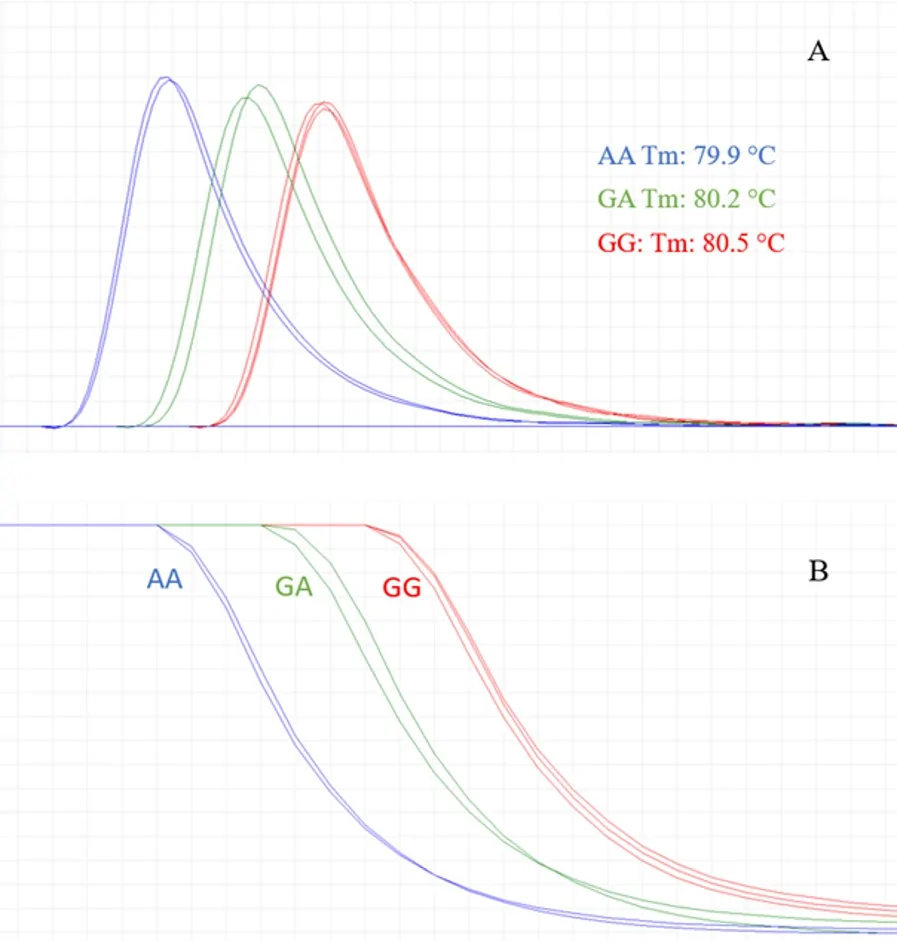
High-resolution melting (HRM) profiles for the SOD1:c.118G>A mutation in Cimarrón Uruguayo dogs. (A) Derivative melting curves showing distinct peaks for AA, GA, and GG genotypes. (B) Normalized HRM curves illustrating clear clustering of the three genotypes.

HRM analysis of the 79 bp amplicon spanning the SOD1:c.118G>A mutation produced clearly distinguishable melting profiles among the three genotypes. Mean melting temperatures were 79.9 °C (AA), 80.2 °C (GA), and 80.5 °C (GG) **(**figure 3**)**, allowing reliable discrimination between homozygous and heterozygous genotypes. A single peak was observed in the melting curve of each sample, confirming the specificity of the primers designed in this study.

### Variant detection in the SOD1 gene

A total of 100 sequences were analyzed. No additional non-synonymous variants were detected apart from the SOD1:c.118G>A substitution in exon 2 **(**Supplementary Material figure 1a). One synonymous substitution (c.285C>T) was identified in exon 4 (Supplementary Material figure 1b). No other sequence variants were detected in the analyzed regions.

### Prediction of the effects of variants on SOD1

**Figure 4:**
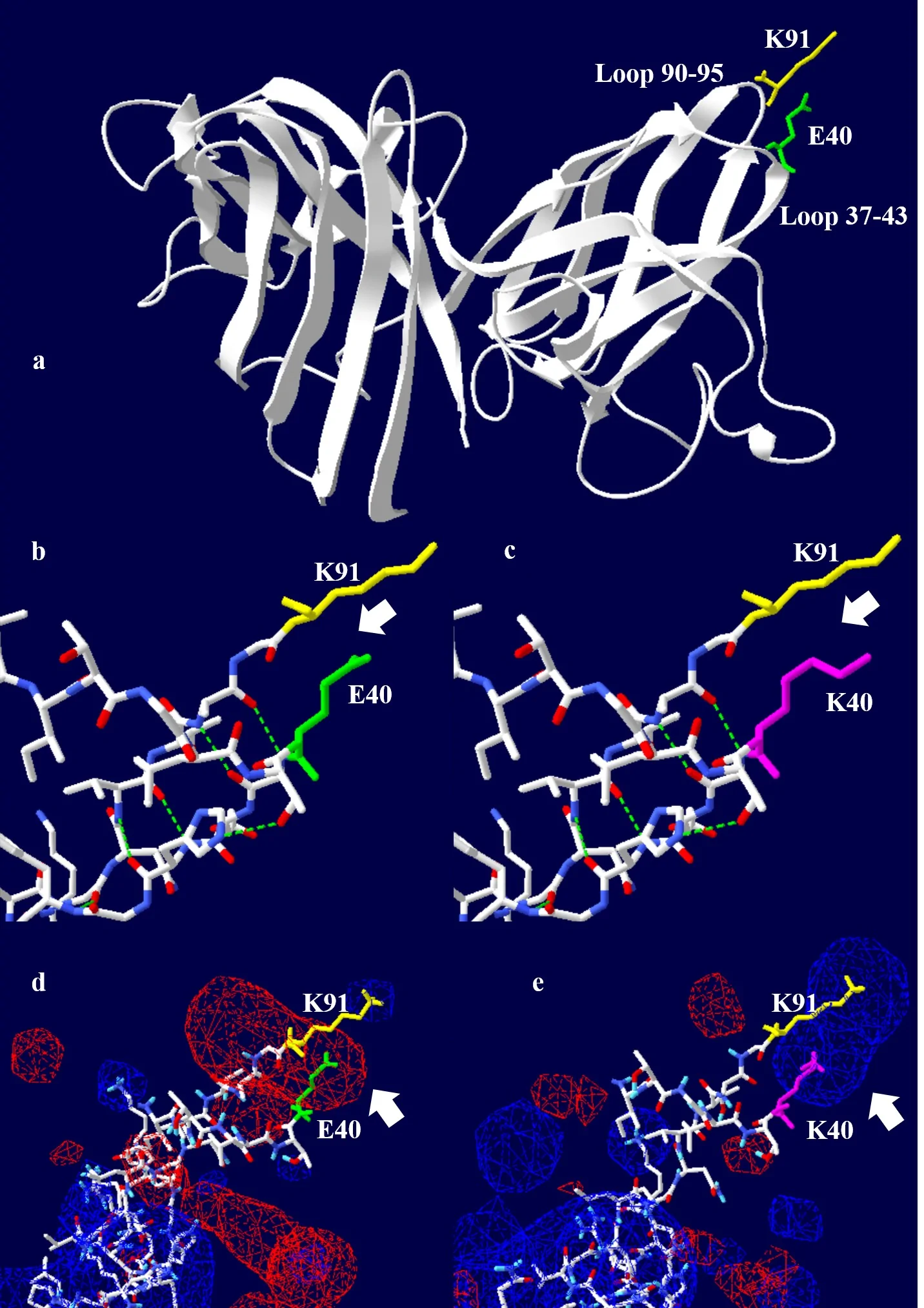
Structural context and local interaction environment of the SOD1 E40K substitution. (a) Overall structure of SOD1 showing the position of residues E40 and K91 in loops 37- 43 and 90 - 95, respectively. (b-c) Local structural environment of residue 40 in the wild-type (E40) and mutant (K40) proteins, showing absence of hydrogen bonds with K91 in both cases. (d-e) Electrostatic potential surface around residues 40 and 91 in the wild-type and mutant proteins, illustrating the charge inversion produced by the substitution; red indicates negative electrostatic potential and blue indicates positive electrostatic potential.

*In silico* structural analysis of the wild-type protein indicated that the glutamic acid residue at position 40 does not form hydrogen bonds with nearby residues (figure 4b). Following the SOD1:c.118G>A substitution, resulting in the replacement of glutamic acid with lysine, the local hydrogen-bonding pattern remained unchanged (figure 4c). Nevertheless, the substitution introduces a charge inversion at the mutation site, replacing a negatively charged residue with a positively charged lysine, thereby modifying the electrostatic landscape of the surrounding region (figure 4d, 4e).

## Discussion

### Population frequency of the SOD1:c.118G>A mutation in the Cimarrón Uruguayo

To our knowledge, this study represents the first analysis of the SOD1:c.118G>A mutation in the Cimarrón Uruguayo breed. The mutant allele was detected within the range of common variants (Goswami et al., 2021), and the genotype distribution did not deviate from Hardy–Weinberg equilibrium, suggesting an absence of strong selective pressures acting on this locus. This is consistent with the late onset and incomplete penetrance of degenerative myelopathy, which allow affected individuals to reproduce before the onset of clinical signs.

Given that Cimarrón Uruguayo dogs may also develop hip dysplasia (https://www.cimarronuruguayo.com/displasia), a condition with overlapping clinical signs, degenerative myelopathy should be considered as a differential diagnosis in cases of chronic and progressive gait impairment.

Reported allele frequencies for the SOD1:c.118G>A mutation vary widely among dog breeds worldwide, ranging from 0.0007 in the West Highland White Terrier (Donner et al., 2023) to 0.94 in the Wire Fox Terrier (Zeng et al., 2014). This wide range reflects substantial variation in demographic history, breeding practices, and effective population size among dog breeds (Awano et al., 2009; Donner et al., 2023; Zeng et al., 2014).

In the present study, the allele frequency estimated for the Cimarrón Uruguayo was 0.177, placing it within the intermediate range reported for multiple dog breeds. Similar frequencies have been reported in the Irish Setter (0.16), Biewer Terrier (0.15), Finnish Lapphund (0.15), and Shiloh Shepherd (0.15) (Donner et al., 2023; Zeng et al., 2014). Comparable values have also been described in some populations of Belgian Malinois (0.17), German Shepherd, and Australian Shepherd (0.14–0.17) (Artigas et al., 2024; Majchrakova et al., 2023; Mataragka et al., 2021).

In contrast, much higher frequencies have been observed in breeds such as the Pembroke Welsh Corgi (0.53–0.79), Boxer (0.42–0.72), and Fox terrier (0.82-0.94), where the mutation approaches fixation in certain populations (Awano et al., 2009; Donner et al., 2023; Zeng et al., 2014). Conversely, in several breeds the mutation occurs at extremely low frequencies, often below 0.01, as reported for breeds such as the West Highland White Terrier, Great Dane, and Samoyed (Donner et al., 2023).

### Genetic drift dynamics of SOD1:c.118G>A in the Cimarrón Uruguayo

In dog breeds with relatively small effective population sizes, genetic drift can significantly influence allele-frequency dynamics. Simulations conducted for the Cimarrón Uruguayo revealed a rapid divergence in allele-frequency trajectories among replicate populations, despite starting from identical conditions (q_0_ = 0.177). Some populations lost the allele entirely, while others showed an increase in allele frequency over time (figure 2a).

A percentile analysis further illustrates the magnitude of this divergence (figure 2b). The lower percentile (P5) reached allele loss within approximately 10–15 generations, and the median trajectory declined to zero by approximately generation 60. In contrast, the upper percentile (P95) steadily increased, exceeding high allele frequencies by generation 100. These findings demonstrate that even identical populations, when subjected solely to genetic drift, can rapidly diverge into distinct allele-frequency distributions. This highlights that in small effective populations such as the Cimarrón Uruguayo, stochastic processes alone can significantly alter the population prevalence of the SOD1:c.118G>A mutation.

When allele frequencies were translated into expected disease prevalence (q^2^), this divergence became even more pronounced. Because degenerative myelopathy follows an autosomal recessive inheritance pattern, small stochastic increases in allele frequency can produce disproportionately large increases in the expected proportion of affected individuals.

The analysis of allele-frequency thresholds revealed that the probability of reaching clinically relevant allele frequencies is highest during an early divergence window, during which a substantial fraction of simulated populations exceeded thresholds such as q ≥ 0.20 or q ≥ 0.25 within a few generations. These results indicate that, depending on the timing of breeding decisions, some subpopulations may rapidly reach clinically relevant allele frequencies purely through genetic drift. This scenario is particularly relevant considering the demographic history of the breed, which is characterized by a reduced number of effective ancestors (n=25) and in which the fact that only 11 individuals explain 50% of the current genetic variability, together with increasing levels of inbreeding observed in recent generations (Martínez et al., 2011).

### HRM-based genotyping of the SOD1:c.118G>A mutation

HRM analysis provides a rapid and cost-effective alternative for SNP genotyping and mutation screening. By detecting differences in the melting behavior of PCR amplicons, HRM allows the discrimination of sequence variants without post-PCR processing. Because the assay is performed in a closed tube, it reduces handling steps, contamination risk, and analysis time compared with conventional gel-based techniques such as PCR-RFLP (Reed et al., 2007; Wittwer et al., 2003).

In the present study, the HRM assay showed clear discrimination among genotypes, demonstrating its potential as a rapid diagnostic approach. The short amplicon size used in this assay (79 bp) may have contributed to the clear separation of melting profiles, since genotype-related differences are often more easily resolved in smaller PCR fragments (Gundry et al., 2003). Consistent with this observation, previous studies have reported improved genotype clustering when HRM targets were reduced to short fragments, such as the 74-bp amplicon described by Słomka et al. (2017). However, the HRM assay proposed here should be validated in a larger number of samples.

Similar HRM approaches have been successfully applied for mutation screening in livestock, including the detection of carriers of complex vertebral malformation and Cholesterol Deficiency in Holstein cattle (Branda Sica et al., 2019, 2022), highlighting their value for large-scale genetic monitoring programs.

### In silico structural impact of the E40K substitution in canine SOD1

Sequence analysis of the *SOD1* gene revealed limited variation within the analyzed coding regions of the ten sequenced individuals. No additional non-synonymous variants were detected beyond the SOD1:c.118G>A mutation, and only a single synonymous substitution was identified in exon 4. Although the small sample size prevents broader conclusions about genetic variability in the breed, the low number of coding changes observed is consistent with the strong functional constraint reported for superoxide dismutase 1, an enzyme essential for the detoxification of superoxide radicals and for maintaining cellular redox homeostasis (Valentine et al., 2005).

SOD1 contains two surface loops (37–43 and 90–95) that, together with residue 144, form an interaction network at one end of the β-barrel that is involved in structural stabilization and protection of aggregation-prone regions. Residue E40 is located in loop 37–43, whereas K91 lies in loop 90–95 (Hart et al., 1998). Wakayama et al. (2022) suggested, based on homology modeling, the existence of a hydrogen bond between these residues. However, inspection of the crystallographic structure analyzed in the present study did not reveal evidence of this interaction, highlighting differences between homology-based models and experimentally determined structures.

Electrostatic interactions, including salt bridges, have been shown to contribute to the structural stability of SOD1, particularly in the region involving residues E40 and K91 (Crown et al., 2019). In this context, the E40K substitution is consistent with a possible mechanism that may promote protein aggregation by disrupting this electrostatic interaction (Kimura et al., 2019). Electrostatic potential mapping performed in the present study revealed that the E40K substitution results in a marked redistribution of charge in the structural region surrounding residues E40 and K91. Such changes in the net charge and electrostatic landscape have been proposed to favor intermolecular interactions and increase aggregation propensity in amyloidogenic proteins (Calamai et al., 2003; Chiti et al., 2002). Previous studies have shown that the E40K mutation does not alter the global fold or structural stability of SOD1, although it reduces the overall net negative charge of the protein (Kimura et al., 2019).

Disruption of the electrostatic interaction between residues 40 and 91 may reduce the stabilizing attraction between the loop regions forming the β-plug of SOD1, thereby increasing local conformational flexibility. This destabilization may facilitate the exposure of residues that are normally buried such as Cys7. Consistent with this, Shino et al. (2019) showed that the E40K substitution increases the accessibility of Cys7, enabling disulfide shuffling reactions in which Cys7 attacks the conserved intramolecular disulfide bond (Cys57–Cys146), thereby leading to the formation of disulfide-linked oligomers of mutant SOD1.

The results of this study highlight the importance of molecular screening of the SOD1:c.118G>A mutation for monitoring degenerative myelopathy in the Cimarrón Uruguayo breed and are consistent with previously proposed structural mechanisms underlying its biological effects. However, the limited number of sequenced individuals and the reliance on in silico structural analyses indicate that further functional and population-level studies are needed.

## Conclusion

The present study provides the first integrated population-level characterization of the SOD1:c.118G>A mutation in the Cimarrón Uruguayo breed. The presence of this variant in the population provides initial insights into its occurrence in the breed. For this reason, it is important to consider degenerative myelopathy as a differential diagnosis in the Cimarrón Uruguayo breed in cases of chronic and progressive gait impairment. In small effective populations such as the Cimarrón Uruguayo, stochastic processes such as genetic drift may influence the future dynamics of this mutation, highlighting the importance of genetic monitoring strategies. In addition, the HRM assay developed in this study offers a rapid and cost-effective approach for genotyping this variant, facilitating its application in genetic screening and monitoring programs. Finally, in silico structural analyses suggest that the E40K substitution may alter the physicochemical environment of SOD1, supporting its biological relevance in the context of degenerative myelopathy.

## Supplementary Material

Supplementary material accompanies this paper.

Supplementary Table 1 Primer sequences for the canine SOD1 gene described by Holder et al. (2014), amplicon size, and annealing temperatures.

Supplementary Figure 1 Representative Sanger sequencing chromatograms of variants detected in the canine SOD1 gene. (a) Non-synonymous substitution c.118G>A detected in exon 2. (b) Synonymous substitution c.285C>T identified in exon 4.

This material is available as part of the online article from https://doi.org/10.29374/2527-2179.bjvm005526
