# Supplementary material for: SOD1:c.118G>A mutation in the Cimarrón Uruguayo dog: prevalence, drift dynamics, and structural effects
Source: Braz J Vet Med. 2026 Sep 11;48:e005526. doi: 10.29374/2527-2179.bjvm005526 (PMC13577606; doi:10.29374/2527-2179.bjvm005526)
Supplement: Supplementary file 1 [file bjvm-48-e005526-suppl001.pdf]

Suppl. Table 1. Primer sequences for the canine *SOD1* gene described by Holder et al. (2014), amplicon size, and annealing temperatures.

**Exón**	**Forward Primer // Reverse Primer**	**Amplicon (bp)**	**Annealing (°C)**
2	F: 5'-AAGTCCATGTTCCTTCCACTTTCTTGTG-3'<br>R: 5'-TTGAGGATTTTCAATGTTTAGGAGTAGC-3'	294	60
3	F: 5'-GTATTTGAGGTTTTAGCAATGGC-3'<br>R: 5'-GCAAGGAATACACCCGTACTGA-53	252	60
4	F: 5'-CTCACAAACTAGCCTGAATCAGTCC-3'<br>R: 5'-GCCCTTCCTAATCTGAACTAAAG-3'	307	60
5a	F: 5'-GCGCTAAGTTATGTTAATGTTCTT-3'<br>R: 5'-AGTTCTCATTACAGGTACTTAAAGCAAT-3'	296	60
5b	F: 5'-AAACATTGTAACCTTAAAAGTGTAATTTG-3'<br>R: 5'-CGTTCAAGCTCTAATGTAGGAGTATGAAG-3'	324	60
HRM*	F: 5'-AAGTGGGCCTGTTGTGGTAT-3'<br>R: 5'-TCCAAACTGATGGACGTGGA-3'	79	60

* Primers designed in this study
