# Supplementary material for: SOD1:c.118G>A mutation in the Cimarrón Uruguayo dog: prevalence, drift dynamics, and structural effects
Source: Braz J Vet Med. 2026 Sep 11;48:e005526. doi: 10.29374/2527-2179.bjvm005526 (PMC13577606; doi:10.29374/2527-2179.bjvm005526)

Suppl. Figure 1. Representative Sanger sequencing chromatograms of variants detected in the canine SOD1 gene. (a) Non-synonymous substitution c.118G>A detected in exon 2. (b) Synonymous substitution c.285C>T identified in exon 4.

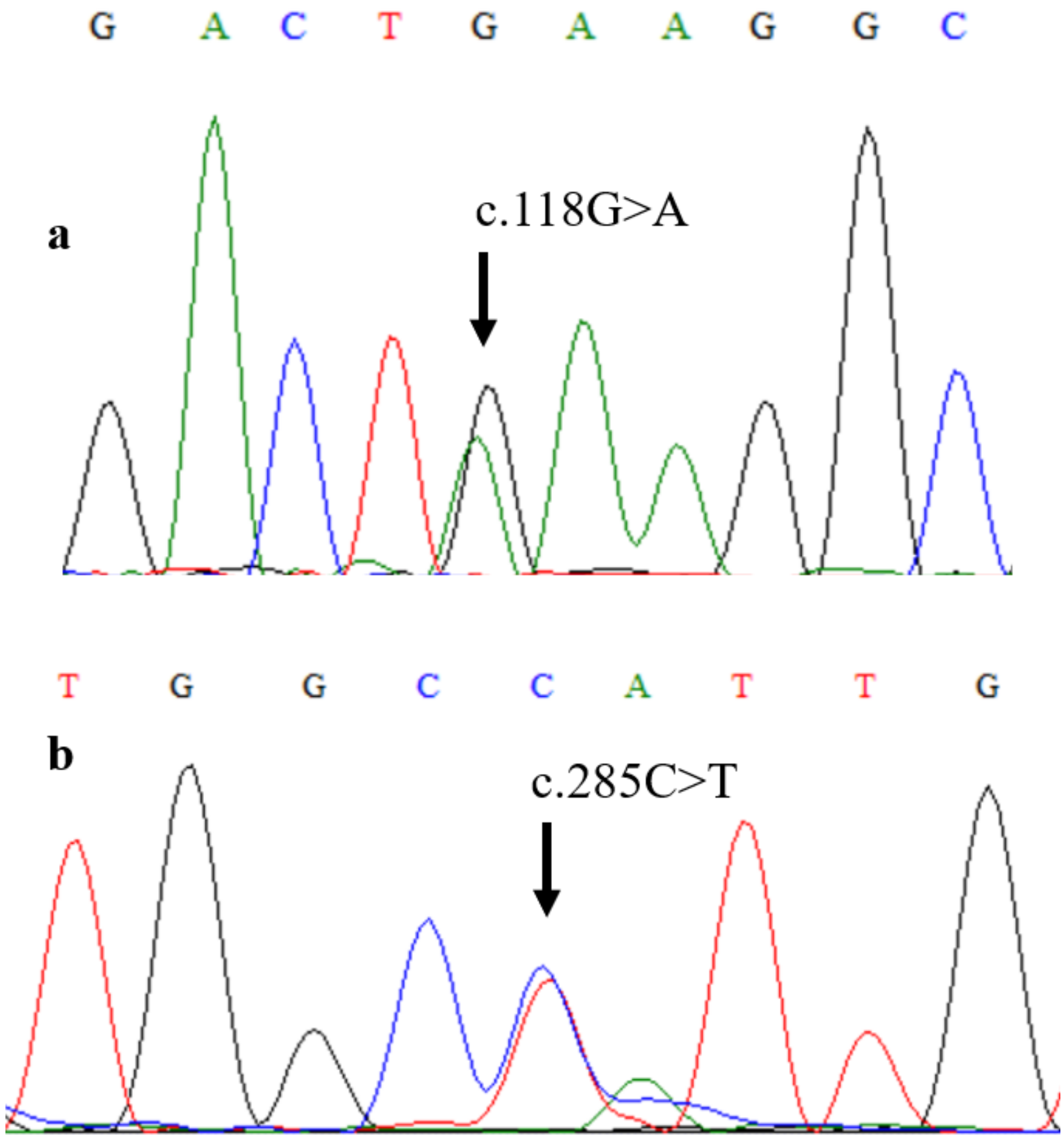

Supplement: Supplementary file 2 [file bjvm-48-e005526-suppl01.pdf]
